# (2-Chloro-6-methyl­quinolin-3-yl)methanol

**DOI:** 10.1107/S1600536810020507

**Published:** 2010-06-05

**Authors:** F. Nawaz Khan, S. Mohana Roopan, Atul Kumar Kushwaha, Venkatesha R. Hathwar, Mehmet Akkurt

**Affiliations:** a Organic and Medicinal Chemistry Research Laboratory, Organic Chemistry Division, School of Advanced Sciences, VIT University, Vellore 632 014, Tamil Nadu, India; b Solid State and Structural Chemistry Unit, Indian Institute of Science, Bangalore 560 012, Karnataka, India; c Department of Physics, Faculty of Arts and Sciences, Erciyes University, 38039 Kayseri, Turkey

## Abstract

The title compound, C_11_H_10_ClNO, is close to being planar (r.m.s deviation for the non-H atoms = 0.026 Å). In the crystal, mol­ecules are linked by O—H⋯O hydrogen bonds, generating *C*(2) chains, and weak C—H⋯π inter­actions and aromatic π–π stacking inter­actions [centroid–centroid distance = 3.713 (3) Å] help to consolidate the structure.

## Related literature

For a related structure and background references, see: Roopan *et al.* (2010 ▶). For the structure of the starting material, see: Khan *et al.* (2009 ▶). For hydrogen-bond motifs, see: Bernstein *et al.* (1995 ▶).
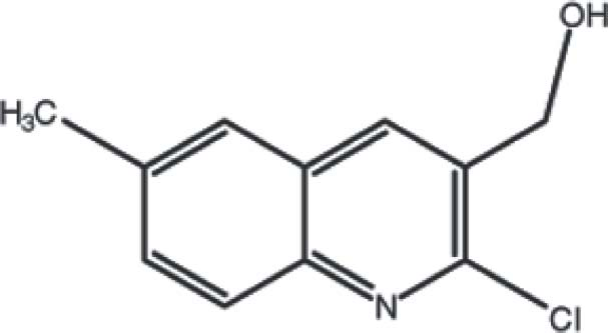


## Experimental

### 

#### Crystal data

C_11_H_10_ClNO
*M* *_r_* = 207.65
Monoclinic, 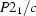

*a* = 14.8091 (17) Å
*b* = 4.6387 (5) Å
*c* = 14.5098 (11) Å
β = 96.594 (9)°
*V* = 990.16 (17) Å^3^
*Z* = 4
Mo *K*α radiation
μ = 0.35 mm^−1^
*T* = 295 K
0.35 × 0.15 × 0.08 mm

#### Data collection

Oxford Xcalibur Eos(Nova) CCD diffractometer
Absorption correction: multi-scan *CrysAlis PRO* RED (Oxford Diffraction, 2009 ▶) *T*_min_ = 0.888, *T*_max_ = 0.973
15485 measured reflections
1721 independent reflections
913 reflections with *I* > 2σ(*I*)
*R*_int_ = 0.136

#### Refinement

*R*[*F* ^2^ > 2σ(*F* ^2^)] = 0.085
*wR*(*F* ^2^) = 0.222
*S* = 0.94
1721 reflections
131 parameters
H atoms treated by a mixture of independent and constrained refinement
Δρ_max_ = 0.41 e Å^−3^
Δρ_min_ = −0.46 e Å^−3^

### 

Data collection: *CrysAlis PRO CCD* (Oxford Diffraction, 2009 ▶); cell refinement: *CrysAlis PRO CCD*; data reduction: *CrysAlis PRO RED* (Oxford Diffraction, 2009 ▶); program(s) used to solve structure: *SHELXS97* (Sheldrick, 2008 ▶); program(s) used to refine structure: *SHELXL97* (Sheldrick, 2008 ▶); molecular graphics: *ORTEP-3* (Farrugia, 1997 ▶); software used to prepare material for publication: *WinGX* (Farrugia, 1999 ▶).

## Supplementary Material

Crystal structure: contains datablocks global, I. DOI: 10.1107/S1600536810020507/hb5471sup1.cif

Structure factors: contains datablocks I. DOI: 10.1107/S1600536810020507/hb5471Isup2.hkl

Additional supplementary materials:  crystallographic information; 3D view; checkCIF report

## Figures and Tables

**Table 1:** Hydrogen-bond geometry (Å, °) *Cg*1 is the centroid of the N1/C1/C6–C9 ring.

*D*—H⋯*A*	*D*—H	H⋯*A*	*D*⋯*A*	*D*—H⋯*A*
O1—H1*O*⋯O1^i^	0.79 (6)	1.93 (6)	2.716 (5)	177 (7)
C10—H10*A*⋯*Cg*1^ii^	0.97	2.73	3.526 (5)	139

Symmetry codes: (i) 
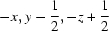
; (ii) 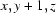.

**Fig. 1.**
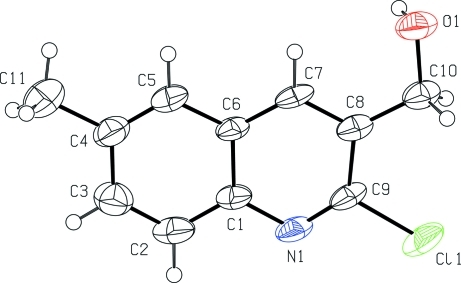
View of the molecular structure of (I), showing 50% probability displacement ellipsoids.

**Fig. 2.**
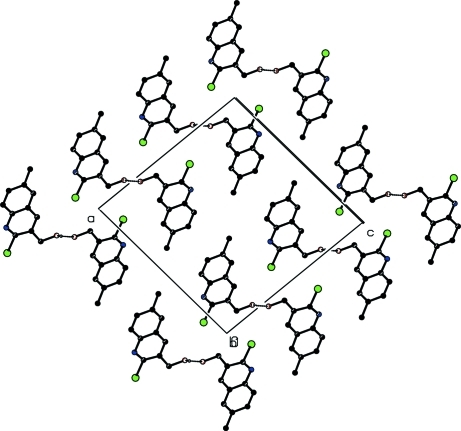
Molecular packing and the hydrogen bonding of (I) viewed down b axis. The H atoms not involved in hydrogen bonds have been omitted for clarity.

## supplementary crystallographic information

### Comment

The importance and general background of the title compound is given in our earler paper (Roopan *et al.*, 2010).

The molecule of the title compound, (I), (Fig. 1), except the hydroxyl and methyl H atoms, is close to planar (r.m.s deviation 0.026 Å).

An intramolecular C—H···O hydrogen bond generates an S(5) ring motif (Bernstein *et al.*, 1995). Molecules of (I) are linked *via* O—H···O hydrogen bonds (Table 1, Fig. 2), an intermolecular C–H···π interactions between the aromatic H atoms of the ethenol substituent and the pyridine (N1/C1/C6–C9) ring of an adjacent molecule (Table 1), and π-π stacking interactions helping to stabilize the crystal structure [*Cg*1···*Cg*2(*x*, 1 + *y*, *z*) = 3.713 (3) Å, where *Cg*1 and *Cg*2 are centroids of the N1/C1/C6–C9 and C1–C6 rings, respectively].

### Experimental

2-Chloro-6-methylquinoline-3-carbaldehyde (206 mg, 1 mmol), sodium borohydride (38 mg, 1 mmol) and catalytic amount of montmorillonite K-10 were taken in an open vessel and the resulting mixture was irradiating at 500 W for 5 min. Ethylacetate was poured into the reaction mixture and filtered off. The filtrated after removal of solvent was subjected to column chromatography packed with silica and ethyl acetate/petroleum ether was used as the eluant. Colourless plates of (I) were grown by solvent evaporation from a solution of the compound in chloroform.

### Refinement

The H atom of the OH group were located in difference map and its positional parameters were refined freely [O1—H1O = 0.79 (6) Å]. The other H atoms were positioned geometrically, with C—H = 0.93–0.97 Å, and refined as riding with *U*_iso_(H) = 1.2 or 1.5 *U*_eq_(C). The value of *R*_int_ [0.136] is greater than 0.12. Since the overall quality of the data may be poor due to the crystal quality.

### Crystal data

**Table:**

C_11_H_10_ClNO	*F*(000) = 432
*M**_r_* = 207.65	*D*_x_ = 1.393 Mg m^−^^3^
Monoclinic, *P*2_1_/*c*	Mo *K*α radiation, λ = 0.71073 Å
Hall symbol: -P 2ybc	Cell parameters from 865 reflections
*a* = 14.8091 (17) Å	θ = 2.7–21.4°
*b* = 4.6387 (5) Å	µ = 0.35 mm^−^^1^
*c* = 14.5098 (11) Å	*T* = 295 K
β = 96.594 (9)°	Plate, colourless
*V* = 990.16 (17) Å^3^	0.35 × 0.15 × 0.08 mm
*Z* = 4	

### Data collection

**Table:**

Oxford Xcalibur Eos(Nova) CCD diffractometer	1721 independent reflections
Radiation source: Enhance (Mo) X-ray Source	913 reflections with *I* > 2σ(*I*)
graphite	*R*_int_ = 0.136
ω scans	θ_max_ = 25.0°, θ_min_ = 3.0°
Absorption correction: multi-scan *CrysAlis PRO* RED (Oxford Diffraction, 2009)	*h* = −17→17
*T*_min_ = 0.888, *T*_max_ = 0.973	*k* = −5→5
15485 measured reflections	*l* = −17→17

### Refinement

**Table:**

Refinement on *F*^2^	Primary atom site location: structure-invariant direct methods
Least-squares matrix: full	Secondary atom site location: difference Fourier map
*R*[*F*^2^ > 2σ(*F*^2^)] = 0.085	Hydrogen site location: inferred from neighbouring sites
*wR*(*F*^2^) = 0.222	H atoms treated by a mixture of independent and constrained refinement
*S* = 0.94	*w* = 1/[σ^2^(*F*_o_^2^) + (0.1359*P*)^2^] where *P* = (*F*_o_^2^ + 2*F*_c_^2^)/3
1721 reflections	(Δ/σ)_max_ < 0.001
131 parameters	Δρ_max_ = 0.41 e Å^−^^3^
0 restraints	Δρ_min_ = −0.46 e Å^−^^3^

### Special details

**Table:**

Geometry. Bond distances, angles *etc*. have been calculated using the rounded fractional coordinates. All su's are estimated from the variances of the (full) variance-covariance matrix. The cell e.s.d.'s are taken into account in the estimation of distances, angles and torsion angles
Refinement. Refinement on *F*^2^ for ALL reflections except those flagged by the user for potential systematic errors. Weighted *R*-factors *wR* and all goodnesses of fit *S* are based on *F*^2^, conventional *R*-factors *R* are based on *F*, with *F* set to zero for negative *F*^2^. The observed criterion of *F*^2^ > σ(*F*^2^) is used only for calculating -*R*-factor-obs *etc*. and is not relevant to the choice of reflections for refinement. *R*-factors based on *F*^2^ are statistically about twice as large as those based on *F*, and *R*-factors based on ALL data will be even larger.

### Fractional atomic coordinates and isotropic or equivalent isotropic displacement parameters (Å^2^)

**Table:**

	*x*	*y*	*z*	*U*_iso_*/*U*_eq_	
Cl1	0.13152 (10)	0.6780 (3)	−0.04947 (8)	0.0717 (6)	
O1	0.0334 (3)	0.9035 (7)	0.2189 (2)	0.0580 (14)	
N1	0.2440 (3)	0.3573 (8)	0.0535 (2)	0.0448 (14)	
C1	0.2832 (3)	0.2406 (10)	0.1357 (3)	0.0409 (16)	
C2	0.3535 (3)	0.0442 (11)	0.1360 (3)	0.0554 (19)	
C3	0.3939 (3)	−0.0710 (12)	0.2179 (4)	0.0583 (19)	
C4	0.3645 (3)	0.0074 (11)	0.3034 (3)	0.0529 (19)	
C5	0.2963 (3)	0.1980 (10)	0.3040 (3)	0.0454 (16)	
C6	0.2534 (3)	0.3238 (9)	0.2222 (3)	0.0369 (16)	
C7	0.1817 (3)	0.5231 (9)	0.2194 (3)	0.0438 (16)	
C8	0.1412 (3)	0.6383 (9)	0.1376 (3)	0.0414 (16)	
C9	0.1799 (3)	0.5394 (10)	0.0572 (3)	0.0454 (16)	
C10	0.0641 (3)	0.8449 (9)	0.1312 (3)	0.0465 (17)	
C11	0.4114 (4)	−0.1220 (14)	0.3929 (4)	0.078 (2)	
H1O	0.014 (4)	0.756 (13)	0.235 (4)	0.0870*	
H2	0.37380	−0.01050	0.08020	0.0670*	
H3	0.44130	−0.20220	0.21680	0.0700*	
H5	0.27670	0.24850	0.36050	0.0550*	
H7	0.16080	0.57900	0.27480	0.0520*	
H10A	0.08270	1.02420	0.10460	0.0560*	
H10B	0.01400	0.76690	0.08970	0.0560*	
H11A	0.37370	−0.09620	0.44180	0.1170*	
H11B	0.46870	−0.02720	0.40930	0.1170*	
H11C	0.42160	−0.32400	0.38410	0.1170*	

### Atomic displacement parameters (Å^2^)

**Table:**

	*U*^11^	*U*^22^	*U*^33^	*U*^12^	*U*^13^	*U*^23^
Cl1	0.0999 (12)	0.0913 (12)	0.0243 (7)	0.0084 (8)	0.0089 (6)	0.0093 (6)
O1	0.085 (3)	0.051 (2)	0.043 (2)	0.0031 (19)	0.0292 (17)	−0.0008 (17)
N1	0.066 (3)	0.048 (2)	0.0223 (19)	−0.001 (2)	0.0139 (17)	−0.0045 (16)
C1	0.048 (3)	0.051 (3)	0.025 (2)	−0.003 (2)	0.0102 (18)	−0.007 (2)
C2	0.061 (3)	0.073 (4)	0.035 (3)	−0.005 (3)	0.018 (2)	−0.007 (2)
C3	0.056 (3)	0.069 (4)	0.051 (3)	0.004 (3)	0.011 (2)	−0.009 (3)
C4	0.056 (3)	0.070 (4)	0.033 (3)	−0.001 (3)	0.007 (2)	0.006 (2)
C5	0.056 (3)	0.056 (3)	0.025 (2)	−0.005 (2)	0.008 (2)	−0.004 (2)
C6	0.050 (3)	0.041 (3)	0.021 (2)	−0.001 (2)	0.0102 (18)	−0.0049 (18)
C7	0.063 (3)	0.053 (3)	0.017 (2)	−0.009 (3)	0.0118 (19)	−0.0055 (19)
C8	0.056 (3)	0.042 (3)	0.028 (2)	−0.007 (2)	0.0125 (19)	−0.003 (2)
C9	0.068 (3)	0.049 (3)	0.020 (2)	−0.005 (3)	0.009 (2)	0.000 (2)
C10	0.070 (3)	0.037 (3)	0.035 (3)	−0.003 (2)	0.017 (2)	−0.003 (2)
C11	0.087 (4)	0.105 (5)	0.041 (3)	0.009 (3)	0.001 (3)	0.006 (3)

### Geometric parameters (Å, °)

**Table:**

Cl1—C9	1.751 (5)	C7—C8	1.375 (6)
O1—C10	1.426 (5)	C8—C9	1.433 (6)
O1—H1O	0.79 (6)	C8—C10	1.485 (6)
N1—C9	1.276 (6)	C2—H2	0.9300
N1—C1	1.376 (5)	C3—H3	0.9300
C1—C2	1.383 (7)	C5—H5	0.9300
C1—C6	1.431 (6)	C7—H7	0.9300
C2—C3	1.376 (7)	C10—H10A	0.9700
C3—C4	1.409 (7)	C10—H10B	0.9700
C4—C11	1.524 (7)	C11—H11A	0.9600
C4—C5	1.343 (7)	C11—H11B	0.9600
C5—C6	1.407 (6)	C11—H11C	0.9600
C6—C7	1.405 (6)		
			
C10—O1—H1O	105 (4)	O1—C10—C8	112.9 (4)
C1—N1—C9	117.8 (4)	C1—C2—H2	120.00
N1—C1—C2	120.3 (4)	C3—C2—H2	120.00
N1—C1—C6	120.8 (4)	C2—C3—H3	120.00
C2—C1—C6	118.9 (4)	C4—C3—H3	120.00
C1—C2—C3	120.7 (4)	C4—C5—H5	119.00
C2—C3—C4	120.8 (4)	C6—C5—H5	119.00
C3—C4—C11	119.5 (4)	C6—C7—H7	119.00
C5—C4—C11	121.6 (4)	C8—C7—H7	119.00
C3—C4—C5	119.0 (4)	O1—C10—H10A	109.00
C4—C5—C6	122.3 (4)	O1—C10—H10B	109.00
C1—C6—C5	118.3 (4)	C8—C10—H10A	109.00
C5—C6—C7	124.3 (4)	C8—C10—H10B	109.00
C1—C6—C7	117.4 (4)	H10A—C10—H10B	108.00
C6—C7—C8	122.3 (4)	C4—C11—H11A	110.00
C7—C8—C10	124.1 (4)	C4—C11—H11B	110.00
C9—C8—C10	122.2 (4)	C4—C11—H11C	110.00
C7—C8—C9	113.8 (4)	H11A—C11—H11B	109.00
Cl1—C9—N1	115.9 (3)	H11A—C11—H11C	110.00
N1—C9—C8	128.0 (4)	H11B—C11—H11C	109.00
Cl1—C9—C8	116.2 (3)		
			
C9—N1—C1—C2	−179.4 (4)	C11—C4—C5—C6	178.8 (5)
C9—N1—C1—C6	−0.6 (7)	C4—C5—C6—C1	1.0 (7)
C1—N1—C9—Cl1	−179.3 (3)	C4—C5—C6—C7	179.8 (5)
C1—N1—C9—C8	−1.1 (7)	C1—C6—C7—C8	−0.5 (6)
N1—C1—C2—C3	179.2 (5)	C5—C6—C7—C8	−179.3 (4)
C6—C1—C2—C3	0.4 (7)	C6—C7—C8—C9	−0.9 (6)
N1—C1—C6—C5	−179.8 (4)	C6—C7—C8—C10	178.7 (4)
N1—C1—C6—C7	1.4 (6)	C7—C8—C9—Cl1	−180.0 (3)
C2—C1—C6—C5	−1.0 (6)	C7—C8—C9—N1	1.8 (7)
C2—C1—C6—C7	−179.8 (4)	C10—C8—C9—Cl1	0.4 (6)
C1—C2—C3—C4	0.2 (8)	C10—C8—C9—N1	−177.8 (4)
C2—C3—C4—C5	−0.2 (7)	C7—C8—C10—O1	−2.5 (6)
C2—C3—C4—C11	−179.4 (5)	C9—C8—C10—O1	177.1 (4)
C3—C4—C5—C6	−0.4 (7)		

### Hydrogen-bond geometry (Å, °)

**Table:**

Cg1 is the centroid of the N1/C1/C6–C9 ring.

**Table:**

*D*—H···*A*	*D*—H	H···*A*	*D*···*A*	*D*—H···*A*
O1—H1O···O1^i^	0.79 (6)	1.93 (6)	2.716 (5)	177 (7)
C10—H10A···Cg1^ii^	0.97	2.73	3.526 (5)	139

Symmetry codes: (i) −*x*, *y*−1/2, −*z*+1/2; (ii) *x*, *y*+1, *z*.
